# ﻿A new species of *Proaphelinoides* Girault (Hymenoptera, Aphelinidae) from China, with a phylogenetic analysis

**DOI:** 10.3897/zookeys.1217.132291

**Published:** 2024-11-07

**Authors:** Yan-yan Jiang, Huifeng Zhao, Ye Chen

**Affiliations:** 1 Hebei Key Laboratory of Animal Diversity, College of Life Science, Langfang Normal University, Langfang, 065000, China Langfang Normal University Langfang China

**Keywords:** Aphelininae, Chalcidoidea, parasitoid wasp, taxonomy

## Abstract

A new species of *Proaphelinoides* Girault, *Proaphelinoideshuangi* Chen & Jiang, **sp. nov.**, is reported from China. A key to all species of the genus is provided. DNA standard barcode COI and partial nuclear ribosomal 28S-D2 from two individuals of *Proaphelinoides* were sequenced, and 28S-D2 rDNA was included in a phylogenetic analysis, confirming *Proaphelinoides* as the sister group to *Aphytis*.

## ﻿Introduction

*Proaphelinoides* Girault is a small genus in Aphelinidae, containing only seven species worldwide, with *P.elongatiformis* from Sri Lanka as the type species (Girault 1917; UCD Community 2023). Other species in the genus include *P.australis* from Australia (Girault 1922), *P.bendovi*Tachikawa (1984) from Guangdong Province of China, and the remaining four species, *P.anomalus*Hayat (1984), *P.chidambaramensis* Manickavasagam & Menakadevi (Menakadevi and Manickavasagam 2012), *P.assamensis*Hayat (2012), and *P.ematus* Hayat &Veenakumari (2016) from India. Their known hosts are diaspidid scales of the genus *Odonaspis* Leonardi (Hemiptera, Diaspididae), which are specialized plant parasites of bamboo (Tachikawa 1984; Si et al. 2019). In China, three species (*P.elongatiformis*, *P.bendovi*, *P.anomalus*) were reported prior to this study (Si et al. 2019).

*Proaphelinoides* is distinguished from related genera by the following combination of characters: body elongate and flattened, antenna 6-merous, pronotum dorsally long, 0.5× as long as mid lobe of mesosoma, medially divided by a suture; propodeum long, more than 3.0× as long as metanotum medially, with posterior margin transverse; linea calva bordered proximally by 1–4 lines of setae and closed posteriorly by a line of setae.

The systematic status of *Proaphelinoides* remains unclear. *Proaphelinoides* was placed in Aphytini in the subfamily Aphelininae (Hayat 1998; Kim and Heraty 2012). The parsimonious tree of Aphelininae based on 50 morphological characters recovered *Proaphelinoides* as the sister group to *Eretmocerus+Marlattiella* (Kim and Heraty 2012). However, *Eretmocerus* is now placed in Eretmocerinae (Heraty et al. 2013; Cruaud et al. 2024). Thus, analyzing the relationship between *Proaphelinoides* and other genera based on molecular data is useful for our understanding the systematic relationship of Aphelininae.

In this study, a new species, *Proaphelinoideshuangi* sp. nov., is described and illustrated. The barcode region of mitochondrial cytochrome oxidase subunit I (COI) and the D2 region of the 28S ribsosomal DNA (28S-D2 rDNA) were sequenced and uploaded to GenBank. A key to all the known species of the genus is provided. In addition, phylogenetic analyses including 26 28S-D2 rDNA sequences together with our new data were carried out to assess the systematic position of the genus.

## ﻿Materials and methods

### ﻿Morphological study

Samples were obtained using a pyrethroid fog generated from a thermal fogger (Swingfog SN50, Germany, Model 2610E, Series 3). Samples fell into collection trays (area of each tray is 1 m^2^) which were suspended 1.5 m above the ground. The collected samples were stored in 100% ethanol at 4 °C in the refrigerator.

Specimens were dissected and mounted in Canada balsam on slides following the method described by Noyes (1982). The methods of photography and measurements following Chen and Chen (2021). Scale bars in the figures are 100 μm except where otherwise indicated. All specimens listed below, including the holotype, are deposited in the collection (LFNU) of the Langfang Normal University, Langfang, China.

Terminology follows Kim and Heraty (2012). The following abbreviations are used in the text: F1–3, flagellomeres 1–3; Gt_1_, Gt_2_ etc., tergites 1, 2, etc. of gaster.

### ﻿Phylogenetic analysis

#### ﻿DNA extraction, amplification, and sequencing

Genomic DNA extraction was from the entire body of female adults using the TIANGEN Genomic DNA Kit (Beijing, China) following the manufacturer’s instructions. COI was amplified using the primers of LCO1490 (5′-GGTCAACAAATCATAAAGATATTGG-3′) (Folmer et al. 1994), HCOout (5′-CCAGGTAAAATTAAAATATAAACTTC-3′) (Carpenter 1999), and the PCR cycling profile followed the procedures in Huangfu et al. (2022). 28S-D2 rDNA was amplified using the primers of 3317F (5′-ACCCGCTGAATTTAAGCATAT-3′) and 4283R (5′-TAGTTCACCATCTTTCGGGTCCC-3′) (Hancock et al. 1988), and the PCR cycling profile followed the procedures in Qin et al. (2022). PCR amplifications were checked by electrophoresis in a 1% agarose gel, and the positive products were sent to Tianyi Huiyuan Biotechnology Co., Ltd (Beijing) for Sanger sequencing using an ABI 3730 automated sequencer. The raw AB1 data was corrected manually in BioEdit v. 7.0.9.0 (Hall 1999).

#### ﻿Phylogenetic analysis

Twenty-six sequences of 28S-D2 rDNA downloaded from GenBank, along with our two new sequences (for GenBank accession numbers see Table 1), representing 11 genera within three subfamilies of Aphelinidae were included in the analysis. Two species of *Coccophagus* (Coccophaginae) were chosen as outgroups. These data were aligned with MAFFT v. 7.5 (Katoh and Standley 2013) and edited manually. Phylogenetic trees were constructed using Bayesian inference (BI) and maximum likelihood (ML). The BI analysis was performed with MrBayes v. 3.2.6 (Ronquist et al. 2012) using the best-fit model GTR+I+G which was selected by jModeltest v. 2.1.7 (Darriba et al. 2012) based on the Akaike information criterion. To ensure the average standard deviation of split frequencies was less than 0.01 in the BI analysis, two million generations were run with sampling every 1000 generations. The ML tree was obtained using RAxML v. 8.2.12 (Stamatakis 2014) with the GTRGAMMA model and the default rapid hill-climbing algorithm; support values were determined using 1000 bootstrap replicates. Both BI and ML trees were visualized and edited in Figtree v. 1.4.4 (Rambaut 2018).

**Table 1.** GenBank accessions for the 28S-D2 rDNA sequences used in the phylogenetic analyses.

Species	GenBank accession no.	Species	GenBank accession no.
*Aphelinusalbipodus*	AY599361	*Eutrichosomella* sp.	AY640319
*Aphelinusasychis*	AY599362	*Mariettacaridei*	MH455947
*Aphelinusparamali*	KF894417	*Marietta* sp.	AY599363
*Aphytismelinus*	JN623554	*Marietta* sp.	KF597646
*Aphytisholoxanthus*	AY635348	*Mariettaleopardina*	AY635301
Aphytisnr.africanus	AY635347	Mariettanr.marchali	AY635300
*Centrodoraacridiphagus*	AY635295	*Neophytisdealbatus*	AY635316
Centrodoranr.penthimiae	AY635297	*Neophytismelanostictus*	AY635317
*Centrodora* sp.	AY599366	Neophytisnr.melanostictus	AY635315
*Coccophagusrusti*	AY599377	*Proaphelinoideshuangi* sp. nov.	PQ038115
*Coccophagus* sp.	AY599376	*Proaphelinoideshuangi* sp. nov.	PQ038116
*Eretmoceruseremicus*	AY599369	*Neophytisdealbatus*	AY635316
*Eretmocerusmundus*	JF820004	*Neophytismelanostictus*	AY635317
*Eretmocerusorchamoplati*	JF750732	*Samariola* sp.	JN623552

## ﻿Results

### ﻿Key to species of *Proaphelinoides* (females)

**Table:** 

1	Fore wing without a group of bristles below the proximal 1/3 of marginal vein; linea calva well defined, proximally bordered by 3 or 4 lines of setae	**2**
–	Fore wing with a group of bristles (Fig. 6); linea calva either absent or bordered by single line of setae	**3**
2	Antenna with scape 4.7× as long as wide; F3 (ventral length) 0.53× width, and with dorsal length equal to width; mid lobe of mesoscutum with 9 setae	***P.ematus* Hayat**
–	Antenna with scape 4× as long as wide; F3 (ventral length) equal to width, and with dorsal length 1.5× as long as width; mid lobe of mesoscutum with 12–14 setae	***P.anomalus* Hayat**
3	Fore wing with linea calva defined by at least a line of setae	**4**
–	Fore wing with linea calva absent or not clearly defined (proximally without a complete line of setae)	**7**
4	Linea calva proximally bordered by 2 lines of setae which become 3 lines in posterior third	***P.assamensis* Hayat**
–	Linea calva proximally bordered by a single line of setae	**5**
5	F3 clearly more than 1.2× as long as wide	***P.australis* Girault**
–	F3 at most 1.2× as long as wide	**6**
6	Metasoma about as long as combined length of head and mesosoma, fore wing with a group of 7 or 8 long bristles below marginal vein, F3 and clava clearly darker than other antennomeres	***P.bendovi* Tachikawa**
–	Metasoma longer than combined length of head and mesosoma, fore wing with 10–14 bristles below marginal vein, antennomeres concolorous	***P.huangi* sp. nov.**
7	Fore wing with a group of 15–17 long bristles below marginal vein	***P.elongatiformis* Girault**
–	Fore wing with 30–35 bristles below marginal vein	***P.chidambaramensis* Manickavasagam & Menakadevi**

#### ﻿ Proaphelinoides huangi

Taxon classificationAnimaliaHymenopteraAphelinidae

Chen & Jiang sp. nov.

48B14866-403B-522E-BA3C-C03522C48AB8

https://zoobank.org/2EE3A0AC-66AC-41EC-B437-4E54733A094A

Figs 1–9

##### Type material.

***Holotype***: China • ♀; Yunnan Province; Xishuangbanna; Mengla County; Menglun Town; 21°53.89'N, 101°16.72'E; 568 m a.s.l.; 12 May. 2019; Z-l Bai, Z-g Chen, C Wang, H Yu leg.; LFNU Proap202405-1 [on slide]. ***Paratypes***: • 8 ♀♀ [5 ♀♀ on slides, Proap202405-2–Proap202405-7; • 2 ♀♀ destroyed for DNA extraction]; same data as holotype; LFNU.

##### Diagnosis.

*Proaphelinoideshuangi* sp. nov. can be distinguished from other species in this genus by the following combination of characters: antenna yellow, fore wing with 10–14 bristles below marginal vein, linea calva proximally bordered by a single line of setae, F3 1.0–1.2× as long as wide, the distance between posterior pair of setae of the mid lobe of mesoscutum more than the distance from a seta to later margin of the plate; the length of Gt_8_ 0.8× as long as the distance between two cercal plates.

##### Description.

**Female.** Body length 0.9–1.2 mm (holotype, 1.2 mm).

***Colour*.** Head with face pale yellow, vertex orange, ocelli red and setae on vertex dark. Mandible brown to dark brown. Antenna yellow. Pronotum pale with brown suffusion. Dorsum of mesosoma yellow, except dark-brown posterior margin of mesoscutellum. Lateral sides of propodeum and mesopleuron brown yellow. Fore wing with following infuscate areas: two small patches below end of submarginal vein, a pale brown band below proximal third of marginal vein, a large area below stigmal vein (Fig. 6). Hind wing mostly hyaline, with slight infuscation below end of marginal vein (Fig. 7). Legs (Fig. 8) pale yellow, with metafemur infuscate dorsally. Gaster with anterior half of Gt_1_, Gt_2_ and Gt_8_ pale, posterior half of Gt_1_ pale brown, remaining tergites dark brown.

**Figures 1–9.**
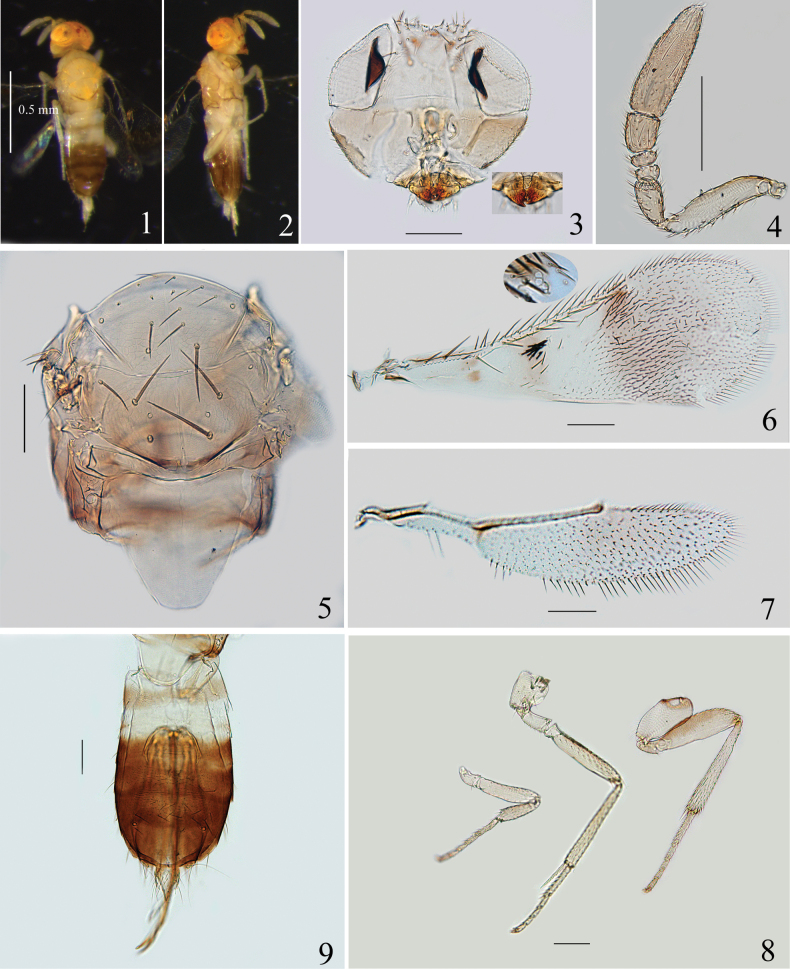
*Proaphelinoideshuangi* sp. nov., holotype female (except Figs 1, 2, 5) **1** body, dorsal view **2** body, lateral view **3** head (inset: mandible) **4** antenna **5** mesosoma **6** fore wing (inset: stigmal vein) **7** hind wing **8** legs (left to right: fore-, mid- and hind-leg) **9** metasoma. Scale bars: 100 μm.

***Head*** 0.8× as high as wide, with weakly reticulate sculpture. Vertex 0.3–0.4× the width of head, with approximately 14 setae. Ocellar triangle with apical angle obtuse. Mandible with three teeth (Fig. 3, inset). Face with 7 setae along inner margin of eyes. Antenna (Fig. 4) with scape 4.2–5.1× as long as wide; pedicle 1.4–1.8× as long as wide; F1 and F2 small and transverse, F1 0.6–0.7× as long as wide, with ventral margin a little longer than F2; F2 0.5× as long as wide; F3 1.0–1.2× as long as wide, with 3 longitudinal sensilla; clava 2.9× as long as wide, a little longer than combined length of pedicle and funicle, with 9–11 longitudinal sensilla.

***Mesosoma*.** Mesoscutum with reticulate sculpture. Mid lobe of mesoscutum 0.8× as long as wide, with approximately 16 setae (Fig. 5), side lobe with 2 setae. Mesoscutellum 0.6× as long as wide, about as long as the mid lobe of mesoscutum, with 2 pairs of setae. The distance between anterior of scutellar setae 1.5× that between posterior pair. Placoid sensilla located in median region of mesoscutellum; distance between sensilla about equal to that between posterior scutellar setae. Metanotum narrow medially. Propodeum long, with median length 0.5× as long as mesoscutellum, with 3 or 4 setae proximal to each spiracle.

***Wings*.** Fore wing (Fig. 6) 2.4–2.9× as long as wide. Costal cell 0.7× length of marginal vein, with 5 or 6 fine setae and 2 long setae distally; submarginal vein with 2 setae; parastigma with 1 seta; marginal vein with 10 setae along anterior margin; postmarginal vein short, about 0.5× as long as stigmal vein; basal cell with 2 setae below end of submarginal vein; 10–14 dark bristles present in a group below proximal third of marginal vein; linea calva proximally bordered by a single line of setae, and closed posteriorly by 1 line of setae. Hind wing (Fig. 7) 3.8–4.6× as long as wide, with longest marginal fringe 0.3× wing width.

***Legs*.** Mesotibial spur about as long as corresponding basitarsus.

***Metasoma*.** Metasoma about 1.5× as long as mesosoma measured from slide-mounted specimens. Gaster (Fig. 9) with setae on each tergite as follows: Gt_1_–Gt_3_ 2 (left side) +2 (right side)), Gt_4_ 3+2, Gt_5_ 12–15 setae, arranged in 3 lines; Gt_6_ 1+1, Gt_7_ 1+1, Gt_8_ with approximately 11 setae. Ovipositor originating from apex of Gt_2_, clearly exerted, and 2.1–2.4× as long as mesotibia. The second valvifer 2.7–3.0× as long as third valvula, the latter 1.5× as long as mesobasitarsus.

**Male.** Unknown.

##### Host.

Unknown.

##### Etymology.

The specific name is derived from the family name of Jian Huang, in honor of his contribution to the taxonomic study of Aphelinidae from China.

##### Distribution.

China (Yunnan).

##### Comments.

This new species resembles *P.bendovi*, and the differences between *P.huangi* sp. nov. and that species are shown in the key. Also, this species is similar to *P.elongatiformis*, and the differences are as follows: (1) fore wing with 10–14 bristles below proximal third of marginal vein (15–17 setae in *P.elongatiformis*); (2) linea calva proximally bordered by a complete line of setae (Fig. 6) (not complete in *P.elongatiformis*); (3) the distance between posterior pair of setae of the mid lobe of mesoscutum more than the distance from a seta to later margin of the plate (less than in *P.elongatiformis*); (4) the length of Gt_8_ 0.8× as long as the distance between two cercal plates (1.2× in *P.elongatiformis*, measurements based on fig. 12 in Hayat 1984). This new species seems difficult to distinguish from *P.australis and P.assamensis*. The species differs from *P.australis* by: (1) F3 1.0–1.2× as long as wide (F3 1.5× as long as wide in *P.australis*); (2) the length of Gt_8_ 0.8× as long as the distance between two cercal plates (Gt_8_ notably longer, 1.8× in *P.australis*); (3) ovipositor 2.1–2.4× as long as mesotibia (2.6× in *P.australis*). From *P.assamensis*, the species can be distinguished by: (1) fore wing with 10–14 dark bristles below proximal third of marginal vein (17–20 in *P.assamensis*); (2) linea calva proximally bordered by a single line of setae (bordered by 2 lines of setae which become 3 lines in posterior third in *P.assamensis*); (3) Gt_8_ 0.8× as long as the distance between two cercal plates (as long as in *P.assamensis*); (3) ovipositor originates from apex of Gt_2_ (ovipositor originates from posterior half of Gt_1_ in *P.assamensis*).

### ﻿Phylogenetic analysis

**Figure 10.**
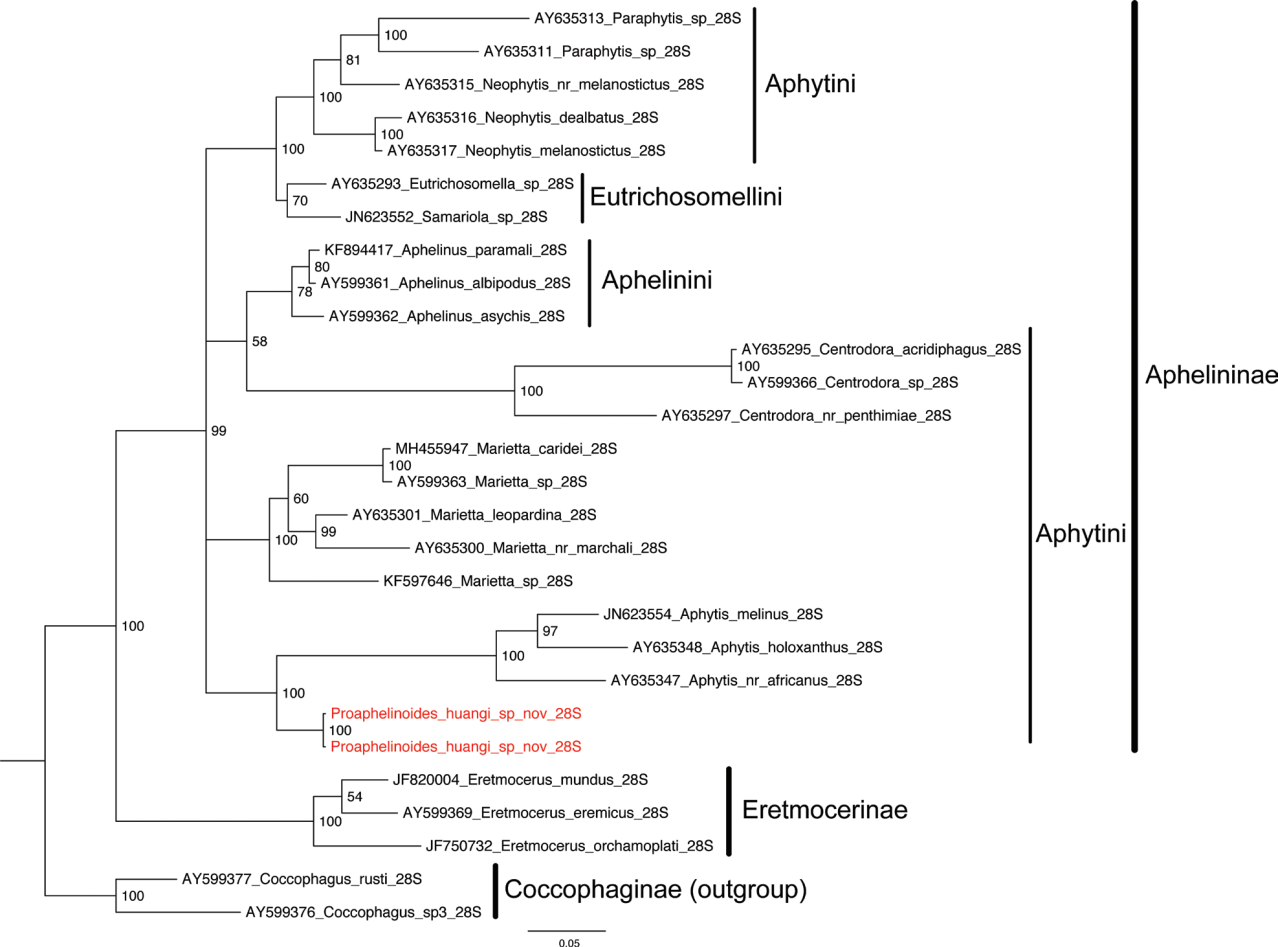
Bayesian phylogenetic tree of Aphelininae based on 28S-D2 rDNA. *Proaphelinoideshuangi* sp. nov. is colored by red.

The phylogenetic relationship between *Proaphelinoides* and other genera is shown in Fig. 10 (BI tree) and Suppl. material 1 (ML tree). *Proaphelinoides* was strongly supported as the sister group to *Aphytis* in both BI and ML analysis with 100 posterior probability and 92% bootstrap support, respectively. The tribe Aphytini was recovered as polyphyletic in both analyses (Fig. 10, Suppl. material 1), which is consistent with the result of Kim and Heraty (2012). In our analysis, this result of Eretmocerinae as the sister group of Aphelininae is congruous with the suggestion of Cruaud et al. (2024). More taxon and gene sampling should be added to further elucidate the systematic relationships within Aphelininae and Aphelinidae.

## Supplementary Material

XML Treatment for Proaphelinoides huangi

## ﻿Additional information

### Author ORCIDs

Huifeng Zhao https://orcid.org/0000-0003-4243-9671

Ye Chen https://orcid.org/0000-0003-0841-6775

### Data availability

All of the data that support the findings of this study are available in the main text or Supplementary Information.

## Supplementary materials

Supplementary material 1 Maximum likelihood phylogenetic tree of Aphelininae based on 28S-D2 rDNA Data type tif https://binary.pensoft.net/file/1169757This dataset is made available under the Open Database License (http://opendatacommons.org/licenses/odbl/1.0/). The Open Database License (ODbL) is a license agreement intended to allow users to freely share, modify, and use this Dataset while maintaining this same freedom for others, provided that the original source and author(s) are credited.Huifeng Zhao
