# Supplementary figures and images for: ﻿A new species of Proaphelinoides Girault (Hymenoptera, Aphelinidae) from China, with a phylogenetic analysis
Source: Zookeys. 2024 Nov 7;1217:263–72. doi: 10.3897/zookeys.1217.132291 (PMC11565181; doi:10.3897/zookeys.1217.132291)

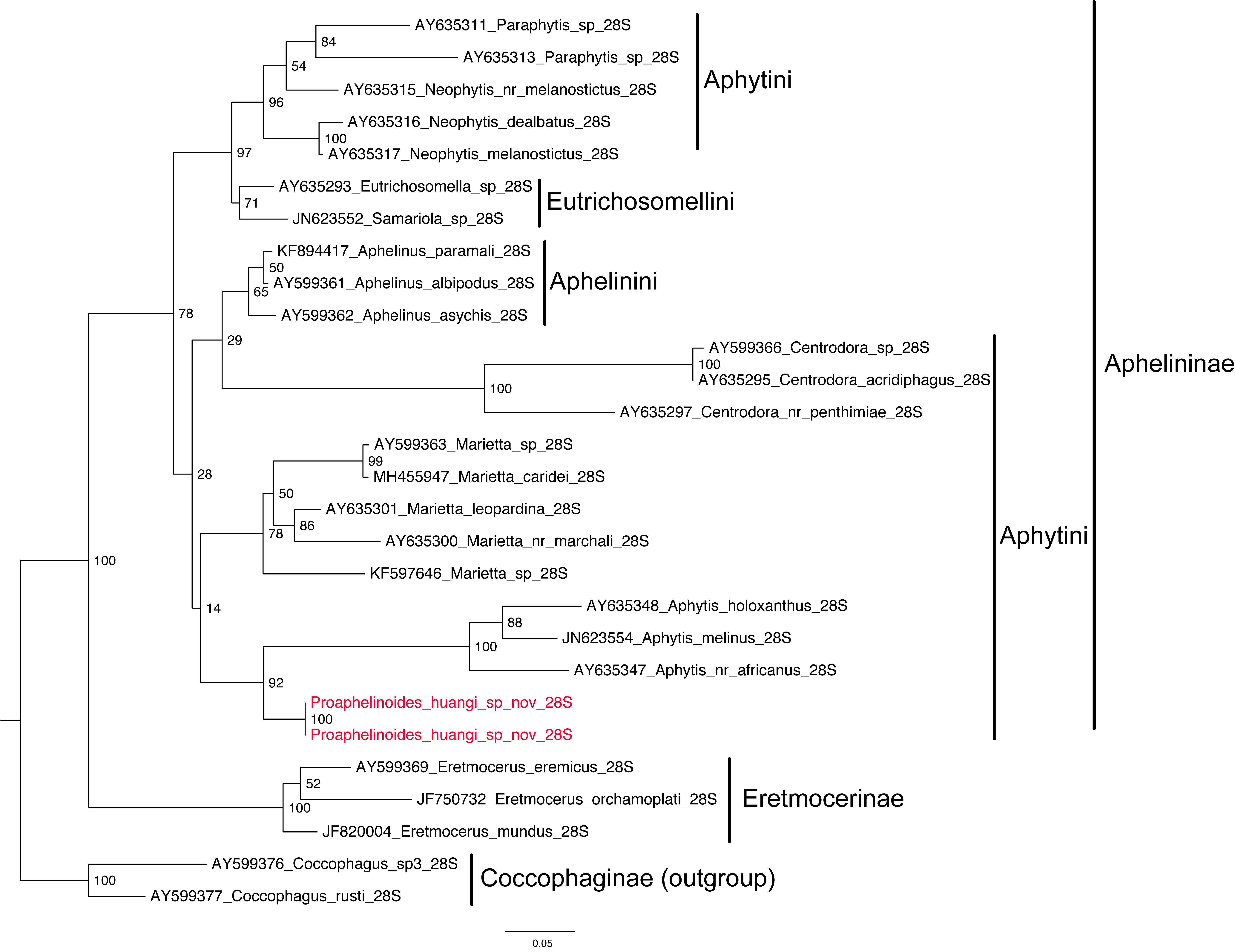

Supplement: Supplementary material 1 — Maximum likelihood phylogenetic tree of Aphelininae based on 28S-D2 rDNA [file zookeys-1217-263_article-132291__-s001.tif]
